# New species and a new record of *Phylloporia* from Benin

**DOI:** 10.1038/s41598-021-88323-3

**Published:** 2021-04-23

**Authors:** Boris Armel Olou, Nourou Soulemane Yorou, Ewald Langer

**Affiliations:** 1 Department of Ecology, Universität Kassel, Heinrich-Plett-Str. 40, Kassel, Germany; 2 Research Unit Tropical Mycology and Plant-Soil Fungi Interactions (MyTIPS), University of Parakou, BP 123, Parakou, Benin

**Keywords:** Fungi, Taxonomy

## Abstract

Species of the wood-decay genus *Phylloporia* (Hymenochaetaceae, Hymenochaetales, Basidiomycota) are widely distributed in the tropics. *Phylloporia* species are, however, morphologically and ecologically diverse, which makes morphology-based species identification challenging. In this study, we re-examined species of *Phylloporia* reported from Benin (West Africa). Using an integrative approach combining morphology, ecology, and phylogenetic analyses, we describe *Phylloporia beninensis* sp. nov. and report *Phylloporia littoralis* for the first time outside of its type locality. *Phylloporia beninensis* sp. nov. is characterized by its annual and imbricate basidiomata, duplex context with a black zone separating the upper context from the lower one, dimitic hyphal system, presence of cystidioles, basidia of 9–12 × 4–5 μm, and subglobose to ellipsoid basidiospores measuring 3–4.6 × 2.1–3.6 μm. Detailed descriptions with illustrations for the new species are provided. With the addition of the new species, 15 *Phylloporia* species are now known to occur in tropical Africa. Our discovery of a new *Phylloporia* species in Benin should stimulate further mycological investigations in tropical African ecosystems to discover other new polypore species. To facilitate further taxonomy studies on tropical African *Phylloporia* taxa, a key to the known tropical African species is provided.

## Introduction

*Phylloporia* Murrill is a widely distributed polypore genus in Hymenochaetaceae (Hymenochaetales, Agaricomycetes, Basidiomycota) typified by *P. parasitica* Murrill^1^. Species of *Phylloporia* occur mainly in the tropics but are also known from higher latitude ecosystems well^2–5^. *Phylloporia* sensu Murrill was characterized by resupinate and annual basidiomata with the ability to grow on the underside of living leaves, monomitic hyphal system, and absence of the setae which characterize most members of the Hymenochaetaceae^1^. Although the genus was erected in 1904, it remained undocumented until Ryvarden^6^ re-examined the type material and recognized four additional species: *P. bibulosa* (Lloyd) Ryvarden, *P. chrysites* (Berk.) Ryvarden, *P. fruticum* (Berk. & M. A. Curtis) Ryvarden, and *P. weberiana* (Bres. & Henn. ex Sacc.) Ryvarden, each sharing the microscopic features characterizing the type species. Subsequently, with the advent of DNA barcoding, *Phylloporia* taxonomy was revisited^7^. Based on phylogenetic analysis inferred from nuc 28S rDNA sequences as well as morphological and anatomical features, Wagner and Ryvarden^7^ demonstrated that *Phylloporia* is monophyletic, with *Fulvifomes* Murrill as a sister genus. From this pioneering molecular work, *Phylloporia* received much more attention, resulting in the current recognition of 61 species worldwide^8–13^. The current morphological concept for *Phylloporia* species includes annual to perennial basidiomata with resupinate, pileate-sessile, or pileate-stipitate habits, homogenous to two-layered context, monomitic to dimitic hyphal system, presence or absence of cystidioles, and subglobose to ellipsoid basidiospores^8, 10, 13, 14^. The presence of setae in *Phylloporia* was not noted until reported by Wu et al.^10^. Ecologically, some *Phylloporia* species are putatively host-specific parasites on living leaves, bushes, branches, and trees^7, 13, 15–17^*,* others are saprotrophs on wood^18–20^, with the trophic status of others still unknown^10^.

Despite the reasonably well-defined generic diagnostic features, *Phylloporia* remains heterogeneous and the species can be difficult to separate morphologically from those of related hymenochaetoid genera. As an example, Douanla-Meli et al.^21^ described a new species from Cameroon as *P. resupinata* Douanla-Meli and Ryvarden, but subsequent molecular phylogenetic analysis placed *P. resupinata* within the hymenochaetoid *Fomitiporella* clade and the species was transferred to that genus^22^.

Currently 14 species have been reported from tropical Africa with eight of these described from type material collected in the region^2, 7, 9, 12, 18, 23^. These species are mainly Central or East African and to our knowledge, only *P. weberiana* was known from West Africa^7^ until Olou et al.^24^ reported two species identified as *Phylloporia* sp. However, since species of *Phylloporia* can be host specific, we have since re-examined both of the species of Olou et al.^24^. Utilizing morphological and molecular phylogenetic analyses, we found that one of these species is *P. littoralis* Decock & Yombiyeni, previously known only from Gabon, while the second is new to science. Here we describe the new species and provide a key to the known tropical African *Phylloporia* species.

## Material and methods

### DNA extraction, amplifications, and sequencing

We extracted DNA from dried specimens using the microwave method^25^. Although previous studies involving *Phylloporia* species have used primarily the nuclear ribosomal large subunit, here we amplified two nuclear ribosomal DNA regions (nrDNA), the internal transcribed spacer (ITS) and the D1–D4 domain of large subunit (LSU). The primer pairs ITS-1F/ITS4^26, 27^ and LR0R/LR5^28^ were used to amplify both target DNA regions. For the polymerase chain reaction (PCR) procedure, the PCR products purification, and Sanger sequencing, we followed Olou et al.^24^. A total of six sequences, composed of two ITS and four LSU, were generated in this study and deposited in GenBank. Table 1 gives the accession numbers for all taxa included in this study.

**Table 1:** Taxa names with collection details and GenBank accession numbers of all sequences of *Phylloporia* spp. used in this study.

Species name	Voucher or strain	Origin	GenBank N°		References
			TS	LSU	
*Fomitiporella resupinata* (cited as *Phylloporia resupinata*)	Cameroon	Douanla-Meli 476	KJ787822	JF712935	^44^
*Fomitiporella sinica*	China	LWZ 20130809-5	KJ787819	KJ787810	^44, 45^
*Fomitiporella tenuissima* (cited as *Inonotus tenuissimus*)	China	Dai 12245	KC456242	KC999902	^45, 46^
*Fomitiporella umbrinella* (cited as *Fomitiporella* sp.)	USA	JV 0509/114	KX181314	KX181336	^45^
*Fulvifomes fastuosus*	Philippines	CBS 213.36	AY558615	AY059057	^7^
*Fulvifomes robiniae *(cited as *Phellinus robiniae* )	USA/Arizona	CFMR 2693	KX065961	KX065995	Unpublished
*Fulvifomes yoroui*	Benin	OAB0097	MN017126	MN017120	^47^
*Inonotus andersonii*	JV1209_66	USA	MN318443	MN318443	Unpublished
*Inonotus hispidus*	92–829		AY624993	AF311014	Unpublished
*Phylloporia afrospathulata*	MUCL 54511			KJ743248	^12^
*Phylloporia afrospathulata*	MUCL 53983			KJ743249	^12^
*Phylloporia alyxiae*	GC 1604-28	Taiwan		LC514408	^10^
*Phylloporia alyxiae*	Chen 1182	Taiwan		LC514407	^10^
*Phylloporia atlantica*	JRF151	Brazil		MG738814	^8^
*Phylloporia atlantica*	JRF142	Brazil		MG738813	^8^
*Phylloporia bibulosa*	Ahmad 27088			AF411824	^7^
*Phylloporia boldo*	CIEFAPcc532	Chile		MK193759	^48^
*Phylloporia boldo*	CIEFAPcc584	Chile		MK193758	^48^
*Phylloporia capucina*	Robledo 1610	Argentina		KJ651919	Unpublished
*Phylloporia* cf. *fruticum*	MUCL 52762	Mexico		HM635668	^49^
*Phylloporia* cf. *fruticum*	ENCB TR&RV858	Mexico		HM635669	^49^
*Phylloporia chrysites*	MUCL 52862	Mexico		HM635667	^49^
*Phylloporia chrysites*	MUCL 52764	Mexico		HM635666	^49^
*Phylloporia clausenae*	Yuan 3528	China		KJ787795	^13^
*Phylloporia clausenae*	Cui8463	China	MH151186	MH165868	^13^
*Phylloporia crataegi*	Dai18133	China	MH151191	MH165865	^50^
*Phylloporia crataegi*	Dai 11016			JF712923	^50^
*Phylloporia cystidiolophora*	Dai13953	China		MG738799	^8^
*Phylloporia cystidiolophora*	Dai13945	China		MG738798	^8^
*Phylloporia dependens*	Cui13763	China	KX242353	MH151190	^11^
*Phylloporia dependens*	Cui 13763		MH151190	KX242353	^11^
*Phylloporia elegans*	FLOR:51179	Brazil		KJ631409	^20^
*Phylloporia elegans*	FLOR:51178	Brazil		KJ631408	^20^
*Phylloporia ephedrae*			MH151184	AF411826	^7^
*Phylloporia flabelliforma*	MUCL 55570	Gabon	NR_154332	KU198350	^23^
*Phylloporia flabelliforma*	MUCL 55569	Gabon	KU198356	KU198349	^23^
*Phylloporia flacourtiae*	Yuan 6362	China		KJ787801	^13^
*Phylloporia flacourtiae*	Yuan 6360	China		KJ787800	^13^
*Phylloporia fontanesiae*	Cui12356	China	MH151188	MH165871	^50^
*Phylloporia fontanesiae*	Li 199			JF712925	^50^
*Phylloporia fulva*	MUCL 54472			KJ743247	^12^
*Phylloporia gabonensis*	MUCL 55572	Gabon	KU198354	KU198352	^23^
*Phylloporia gabonensis*	MUCL 55571	Gabon	NR_154331	KU198353	^23^
*Phylloporia gutta*	Dai16070	China	MH151183	MH165863	^50^
*Phylloporia gutta*	Dai 4197			JF712927	^50^
*Phylloporia hainaniana*	Dai 9460			JF712928	^50^
*Phylloporia homocarnica*	Yuan 5766	China		KJ787804	^13^
*Phylloporia homocarnica*	Yuan 5750	China	MH151195	KJ787803	^13^
*Phylloporia inonotoides*	MUCL 54468	China		KJ743250	^12^
*Phylloporia lespedezae*	Dai17065	China	MH151179	KY242602	^16^
*Phylloporia lespedezae*	Dai17067	China	MH151180	KY242603	^16^
*Phylloporia littoralis*	MUCL: 56145	Gabon		KY349141	^17^
*Phylloporia littoralis*	MUCL: 56144	Gabon		KY349140	^17^
*Phylloporia lonicerae*	Dai17900	China	MH151175	MG738802	^15^
*Phylloporia lonicerae*	Dai17899	China	MH151174	MG738801	^15^
*Phylloporia lonicerae*	Dai17898	China	MH151173	MG738800	^15^
*Phylloporia manglietiae*	Cui 13709	China	MF410324	KX242358	^11^
*Phylloporia minuta*	FURB 55088	Brazil		NG_064479	^51^
*Phylloporia minutipora*	Dai16172	China		MH165873	Unpublished
*Phylloporia minutispora*	Ipulet 706			JF712929	^50^
*Phylloporia minutispora*	MUCL 52865	Democratic Republic of the Congo		HM635671	^49^
*Phylloporia montana*	BDNA2409	Brazil		MG738811	^8^
*Phylloporia montana*	BDNA2388	Brazil		MG738810	^8^
*Phylloporia mori*	Taiwan	Wu 1105-2		LC514412	^10^
*Phylloporia mori*	Taiwan	Wu 1105-3		LC514413	^10^
*Phylloporia mori*	Wu 1105-3	Taiwan		LC514413	^10^
*Phylloporia mori*	Wu 1105-2	Taiwan		LC514412	^10^
*Phylloporia murrayae*	Wu 1404-5	Taiwan		LC514410	^10^
*Phylloporia murrayae*	Wu 1404-4	Taiwan		LC514409	^10^
*Phylloporia nandinae*	Dai 10625			JF712931	^50^
*Phylloporia nandinae*	Dai 10588			JF712930	^50^
*Phylloporia nodostipitata*	FLOR:51175	Brazil		KJ631413	^20^
*Phylloporia nodostipitata*	FLOR:51173	Brazil	KJ639057	KJ631412	^20^
*Phylloporia nouraguensis*	MUCL/FG-11-409	Guyana		KC136224	^22^
*Phylloporia nouraguensis*	MUCL/FG-11-404	Guyana		KC136223	^22^
*Phylloporia oblongospora*	Zhou179		MH151197	JF712932	^50^
*Phylloporia oreophila*	CUI2219	China	MH151196	JF712933	^50^
*Phylloporia oreophila*	Cui 9503	China		JF712934	^50^
*Phylloporia osmanthi*	Yuan 5655	China		KF729938	^19^
*Phylloporia parasitica*	Leif Ryvarden 19843	Argentina	KU198361		^23^
*Phylloporia pectinate*	R. Coveny 113			AF411823	^7^
*Phylloporia pendula*	Cui 13691	China		KX242357	^11^
*Phylloporia pendula*	Cui 13876	China		KX901670	^11^
*Phylloporia perangusta*	Dai18139	China	MH151169	MG738803	^8^
*Phylloporia pseudopectinata*	Cui 13749	China		KX242356	^11^
*Phylloporia pseudopectinata*	Cui 13746	China		KX242355	^11^
*Phylloporia pulla*	Dai 9627	China		KU904469	^41^
*Phylloporia pulla*	Cui 5251	China		KU904468	^41^
*Phylloporia radiata*	LWZ 20141122-5			KU904470	^41^
*Phylloporia rattanicola*	Dai18235	China	MH151172	MG738808	^8^
*Phylloporia rattanicola*	Dai18233	China		MG738807	^8^
*Phylloporia resupinata*	Douanla-Meli 476	Cameroon	KJ787822	JF712935	^50^
*Phylloporia ribis* (cited as *Phellinus ribis*)		82-828		AF311040	^52^
*Phylloporia rinoreae* (cited as *Phylloporia* sp.)	MUCL: 57328	Gabon		MN243146	^9^
*Phylloporia rinoreae* (cited as *Phylloporia* sp.)	MUCL: 56283	Gabon		MN243144	^9^
*Phylloporia rubiacearum*	Chen 3584	Taiwan		LC514417	^10^
*Phylloporia rubiacearum*	Chen 3583	Taiwan		LC514416	^10^
*Phylloporia rzedowskii*	MUCL 52860	Mexico		HM635674	^49^
*Phylloporia rzedowskii*	MUCL 52859	Mexico		HM635673	^49^
*Phylloporia solicola*	JRF145	Brazil		MG738815	^8^
*Phylloporia* sp.	OAB0107	Benin		MW244097	This study
*Phylloporia* sp.	OAB0142	Benin	MW244094	MW244099	This study
*Phylloporia* sp.	OAB0204	Benin	MW244095	MW244098	This study
*Phylloporia* sp.	OAB0511	Benin		MW244096	This study
*Phylloporia* sp.	FLOR:51258	Brazil		KJ631406	unpublished
*Phylloporia* sp.	FLOR:51239	Brazil		KJ631407	unpublished
*Phylloporia* sp.	Robledo 1220	Argentina		KC136225	^22^
*Phylloporia* sp.	MUCL:KE_16_107	Kenya		KY349147	^17^
*Phylloporia* sp.	MUCL CU05_249			KJ743282	^12^
*Phylloporia* sp.	MUCL/FG-11-506	Guyana		KC136227	^22^
*Phylloporia* sp.	MUCL/FG-11-462	Guyana		KC136228	^22^
*Phylloporia* sp.	MUCL 53433	Mexico		KC136231	^22^
*Phylloporia* sp.	MUCL 52864	Ecuador		KJ743276	^12^
*Phylloporia* sp.	MUCL 45062	Cuba		KJ743284	^12^
*Phylloporia* sp.	MUCL 43733	Cuba		KJ743278	^12^
*Phylloporia* sp.	LWZ 20150531-14	China		KU904466	^41^
*Phylloporia* sp.	Dai 9257	China		KU904464	^41^
*Phylloporia* sp.	ISA007	Brazil		KJ743265	^12^
*Phylloporia* sp.	MUCL 54295	Brazil		KJ743269	^12^
*Phylloporia* sp.	ISA_352	Brazil		KJ743267	^12^
*Phylloporia* sp.	MUCL FG12_523	French Guiana		KJ743260	^12^
*Phylloporia* sp.	MUCL FG12_522	French Guiana		KJ743259	^12^
*Phylloporia* sp.	MUCL FG11_506	French Guiana		KJ743258	^12^
*Phylloporia* sp.	MUCL FG13_722	French Guiana		KJ743264	^12^
*Phylloporia* sp.	MUCL FG13_721	French Guiana		KJ743263	^12^
*Phylloporia spathulata*	Chay456			AF411822	^7^
*Phylloporia splendida*	Dai6282	China		MG738805	^8^
*Phylloporia splendida*	Cui8429	China		MG738804	^8^
*Phylloporia terrestris*	Yuan 5738	China		KC778784	^19^
*Phylloporia terrestris*	He2359	China	MH151189	MH165869	^19^
*Phylloporia tiliae*	Yuan 5491	China		KJ787805	^13^
*Phylloporia ulloai*	MUCL 52867	Mexico		HM635678	^49^
*Phylloporia ulloai*	MUCL 52866	Mexico		HM635677	^49^
*Phylloporia weberiana*	Dai 9242			JF712936	^50^
*Phylloporia yuchengii*	YG 051	Uzbekistan		KM264325	^53^

### Sequence alignment and species delimitation

To place our newly generated sequences accurately in the phylogenetic tree, we aligned them in addition to 126 LSU sequences retrieved from GenBank and used by previous studies on *Phylloporia*^29^. Sequences were aligned using the online mode of MAFFT version 7^30^, with the algorithm FFT-NS-i as the most suitable. The resulting multiple sequences alignment was checked in Geneious 5.6.7 (https://www.geneious.com)^31^, where the ends rich in gaps were manually trimmed. Further, the multiple sequences alignment was inspected and some bases were manually adjusted using AliView^32^. Two model-based methods for species delimitation namely the Automated Barcode Gap Discovery (ABGD)^33^ and the Poisson Tree Process (PTP)^34^ were performed. The ABGD analysis detect potential barcode gap and use the identified barcode gap to sort the datasets into a hypothetical species. This analysis was performed on ABGD web interface using the Jukes-Cantor (JC69) and Kimura two-parameter (K2P). The relative gap width was set to 1.0 because if the gap is too large, the model will sort the dataset into a single species. We kept all other parameters as default. Like the ABGD method, the PTP is another species delimitation method that inferred putative species boundaries on a given phylogenetic input tree. To run the PTP analysis, we first built a single phylogenetic tree using IQ-tree 1.6.12 (http://www.iqtree.org/) in command line mode. The resulted tree without annotations in Newick format was used as the input tree to run the PTP analysis on a web server (http://species.h-its.org/ptp/) for 500,000 generations and 25% were discarded as burn-in. To compare both species delimitation models to the phylogenetic analysis, Maximum likelihood (ML) analysis under the Ultrafast Bootstrap with 5000 replicates was performed on the dataset using IQ-tree 1.6.12 (http://www.iqtree.org/) in command line mode with TM3 + F + I + G4 as the best substitution model selected using the command TESTONLY.

### Phylogenetic analyses

For phylogenetic analyses, 73 sequences from the LSU region out of the 126 sequences previously used to inform species delineation in *Phylloporia* were selected and aligned with the 4 newly generated sequences in this study. In addition, 34 sequences from the ITS region including the type material of the genus were downloaded from GenBank and aligned together with the sequences newly generated in this study. *Inonotus andersonii* (Ellis & Everh.) Nikol. and *I. hispidus* (Bull.) P. Karst. were chosen as outgroup for both regions. Each region was aligned separately using the online mode of MAFFT version 7^30^, with the algorithm L-INS-i. The multiple sequences alignments were checked and concatenated in Geneious 5.6.7 (https://www.geneious.com)^31^.

Given the gap in terms of number of sequences between the ITS and LSU regions (36 vs. 77), the concatenated alignment was considered as a single region and the best-fit evolutionary model was estimated as GTR + I + G using IQ-tree 1.6.12 (http://www.iqtree.org/) and the command TESTONLY. Following this substitution model, two phylogenetic tree inference methods, ML and Bayesian inference (BI) were performed. The ML was run using RAxML 8.2.10^35^ under standard bootstrap at the Cipres Science Gateway V.3.3^36^. The BI was executed using MrBayes 3.2.7 in command line mode (https://github.com/NBISweden/MrBayes)^37^ for five million generations until the standard deviation of split frequencies reached 0.01. Chain convergence was determined using Tracer.v1.7.1 (http://tree.bio.ed.ac.uk/software/tracer/) and the first 25% (5000) trees was discarded as burn-in. The remaining trees were used to build the consensus tree using the Phylogenetic Tree Summarization (SumTrees) program within DendroPy 4.3.0. (https://github.com/jeetsukumaran/DendroPy)^38^. The topology of the ML tree was better resolved than that of BI, so the ML tree was targeted. To add the posterior probabilities (PP) of BI on the ML tree, the Phylogenetic Tree Summarization (SumTrees) program within DendroPy 4.3.0. (https://github.com/jeetsukumaran/DendroPy)^38^ was used. Then, the bootstrap values were added to the ML best tree already having the posterior probabilities using IQ-tree^39^. The resulting tree with (PP/BS) is presented in Fig. 3 and the support values ≥ 80% of PP and ≥ 70% of BS are indicated on each node. Alignment and phylogenetic tree generated in the study are deposited in TreeBASE: http://purl.org/phylo/treebase/phylows/study/TB2:S27303.

### Morphological examination

Morphological descriptions were based on dried herbarium specimens. Macro-morphological characters were described with the aid of a stereomicroscope Leica EZ4 while microstructures were described using a Leica DM500 light microscope. For the microstructures, fine sections through the basidiomata were prepared for observation using a razor blade under a stereomicroscope and mounted in distilled water and 5% aqueous solution of potassium hydroxide (KOH) mixed with 1% aqueous solution of phloxine. Melzer’s reagent (to test for dextrinoid or amyloid reactions) and cotton blue (to test for cyanophilic reaction) were used and then examined at a magnification of 1000×. Leica Application Suite EZ V.3.4 software (Leica Microsystems Ltd., Switzerland) was used to capture images from the microscope. Measurements from captured images were done with the software “Makroaufmaßprogramm” from Jens Rüdigs (https://ruedig.de/tmp/messprogramm.htm) and analyzed with the software “Smaff” version 3.2^40^.

## Results

### Species delimitation

**Figure 1:**
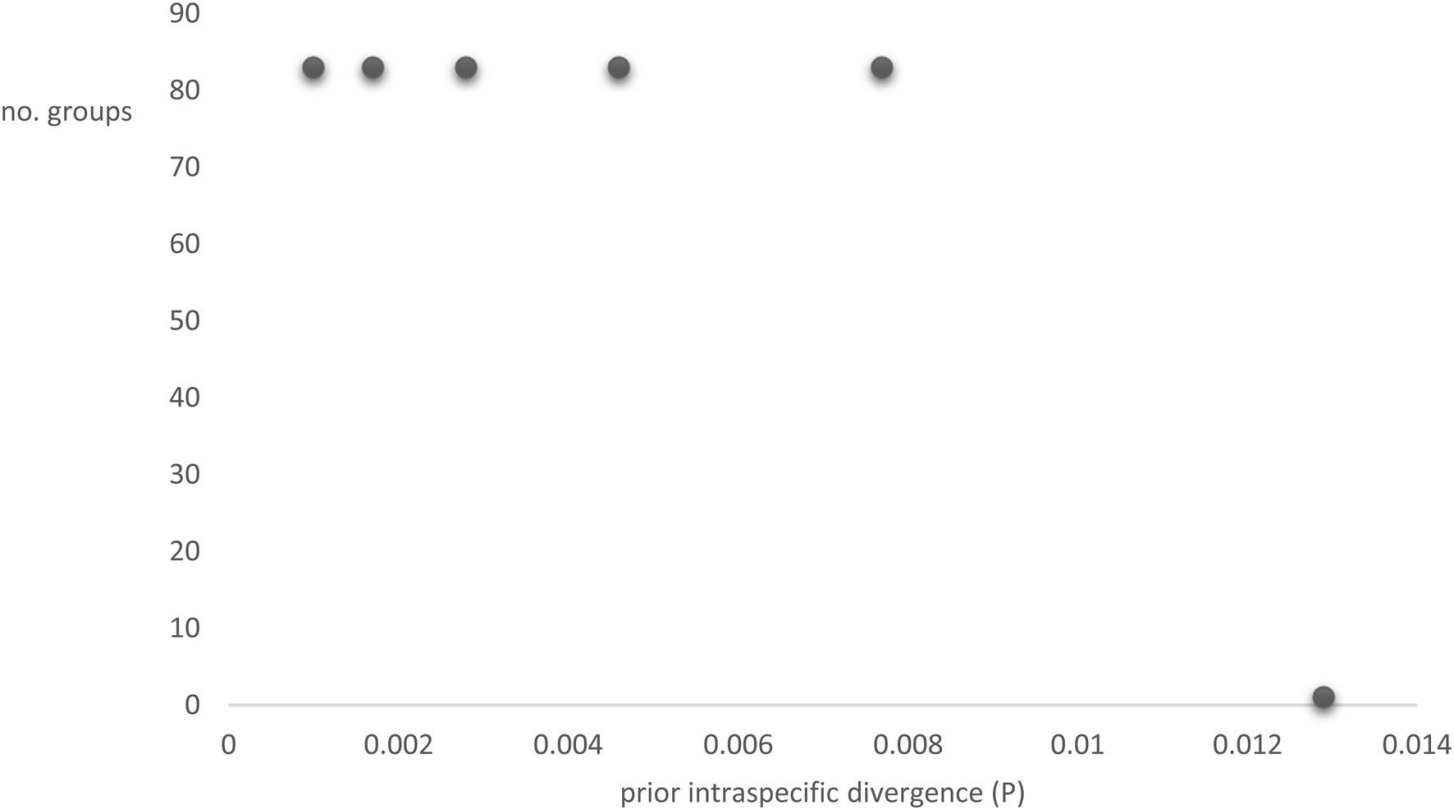
LSU data partition from Automatic Barcode Gap Detection (ABGD).

The ABGD method with parameters JC69 and K2P gave identical results and partitioned the LSU dataset into 6 partitions. The first five partitions with interspecific priority divergence ranging from P = 0.001 to P = 0.0077 contained 83 groups each while the sixth partition with interspecific priority divergence P = 0.0129 contained only one group (Fig. 1). Each group within each partition represented a hypothetical species with one or several sequences (Supplementary Table S1). Given the congruence between the first five partitions (83 groups in each), we have chosen one of them as the one that reflects well our dataset. Thus, all 130 sequences contained in our dataset represent 83 hypothetical species (Supplementary Table S1). The four newly generated LSU sequences in this study were sorted into two groups. The new sequence named *Phylloporia* sp. OAB0204 clustered together with other sequences of *P. littoralis* retrieved from GenBank. The other sequences (*Phylloporia* sp. OAB0107, *Phylloporia* sp. OAB0142, and *Phylloporia* sp. OAB0511) grouped together to form a distinct group (Supplementary Table S1).

**Figure 2:**
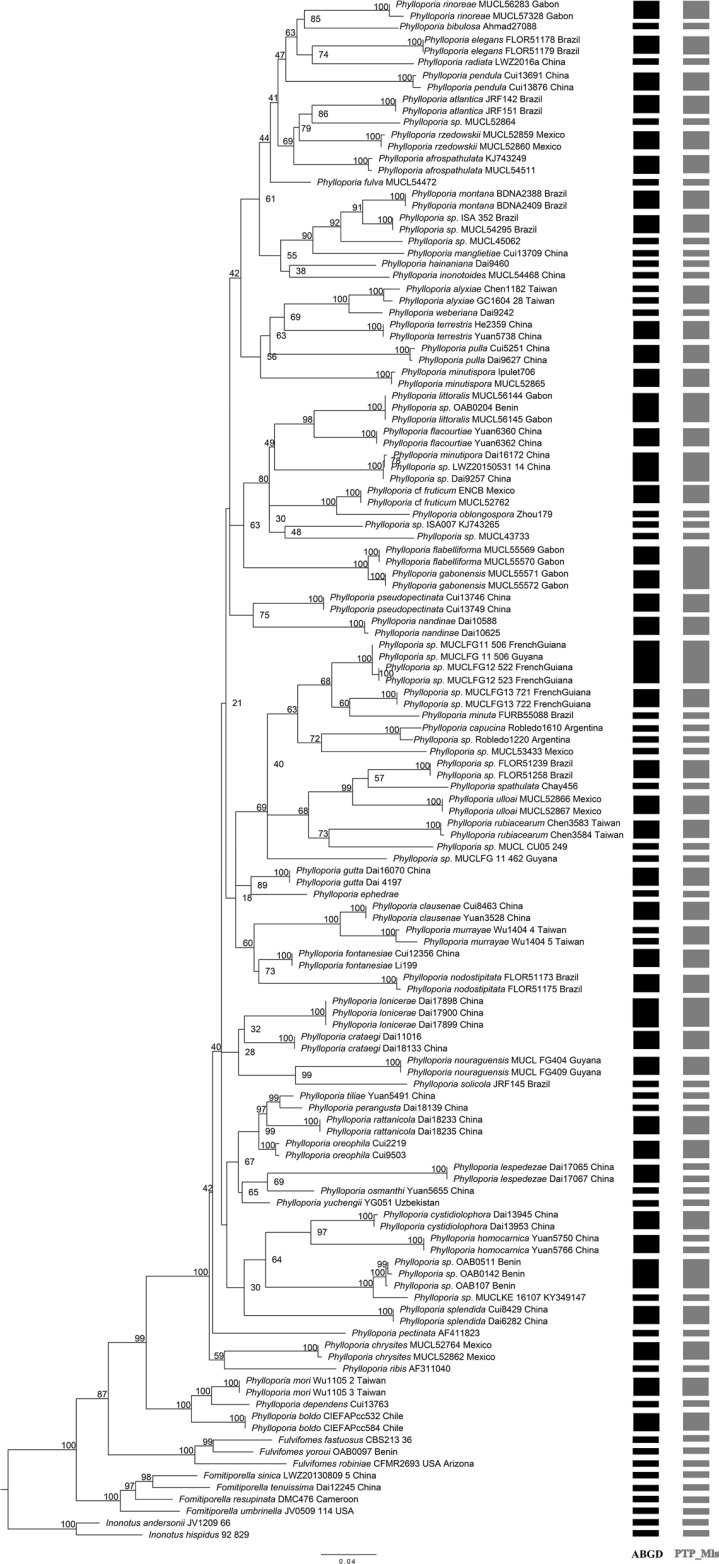
Maximum likelihood tree of the LSU dataset of *Phylloporia* with rapid bootstrap values and species delimitation as recovered in ABGD and PTP analyses. The sequence names are followed by voucher or strain number and country of origin.

The PTP species delimitation estimated that the number of species in LSU dataset was between 82 and 109, with the Mean of 97 species. The PTP species delimitation was supported by the maximum likelihood solution (PTP_Mls) and the Bayesian solution (PTP_Bs). Both solutions gave two different results in terms of the number of estimated species. The PTP_Mls yielded into 82 putative species (Supplementary Table S2) while PTP_Bs gave 100 putative species (Supplementary Table S3). Although the PTP_Mls and PTP_Bs yielded different results, the newly generated sequences formed two distinct species and are grouped identically in both outcomes (Supplementary Table S2, 3). Since species delimitation with PTP_Mls and PTP_Bs gave same results for our newly generated sequences with good support values, and considering the ML tree and ABGD results, we chose the results from PTP_Mls as the most suitable for our dataset. The Fig. 2 presents the ML tree with the putative species as found with ABGD and PTP_Mls.

### Phylogenetic analyses

**Figure 3:**
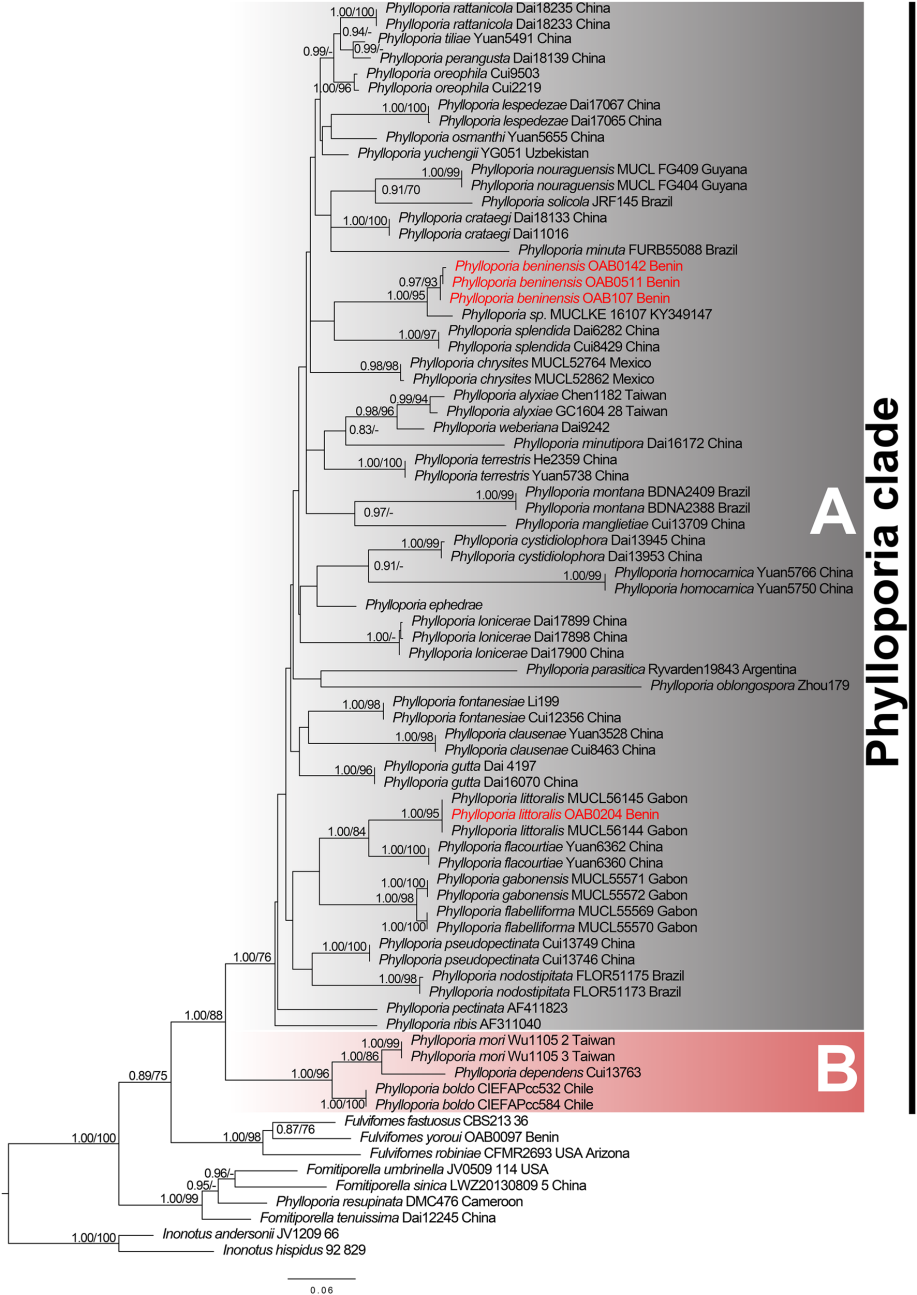
Bayesian analysis (BY) and maximum likelihood (ML) analyses of the combined ITS-LSU dataset of *Phylloporia*. Branch support values given as PP/BS. Newly generated sequences are highlighted in red. The sequence names are followed by voucher or strain number and country of origin.

The combined ITS-LSU alignment contained 78 sequences with 2397 characters, of which 711 were parsimony-informative, 277 singleton sites, and 1409 constant sites. Four well supported major clades namely *Fomitiporella* (PP = 1.00/BS = 99), *Fulvifomes* (PP = 1.00/BS = 98), *Inonotus* (PP = 1.00/BS = 100), and *Phylloporia* (PP = 1.00/BS = 88) were recovered from the phylogenetic analyses inferred from the ITS-LSU (Fig. 3). *Phylloporia* appeared as a well-supported monophyletic clade, which split into two well-supported groups, here named A and B (Fig. 3). Group A (PP = 1.00/BS = 76) contained the sequences of the most *Phylloporia* species, including the generic type (*P*. *parasitica*), while group B (PP = 1.00/BS = 96) consisted of just three species of *Phylloporia*. The newly generated sequences nested within group A. The sequence OAB0204 clustered together with *P*. *littoralis* as it was found in the species delimitation analyses with high support (PP = 1.00/BS = 95). Sequences OAB0107, OAB 0142, and OAB0511 formed a distinct well-supported lineage (PP = 0.97/BS = 93) and had as a sister lineage an unidentified *Phylloporia* species from Kenya with high support (PP = 1.00/BS = 95). Since the sequences OAB0107, OAB 0142, and OAB0511 grouped together and had always formed a distinct lineage in all analyses (Figs. 2, 3; Supplementary Table S1–3), we proposed here as a new species and performed a detailed anatomical–morphological description on these specimens.

### Taxonomy

*Phylloporia beninensis* Olou & Langer, sp. nov.

*MycoBank No. MB839326*

Figures 4, 5, 6

**Figure 4:**
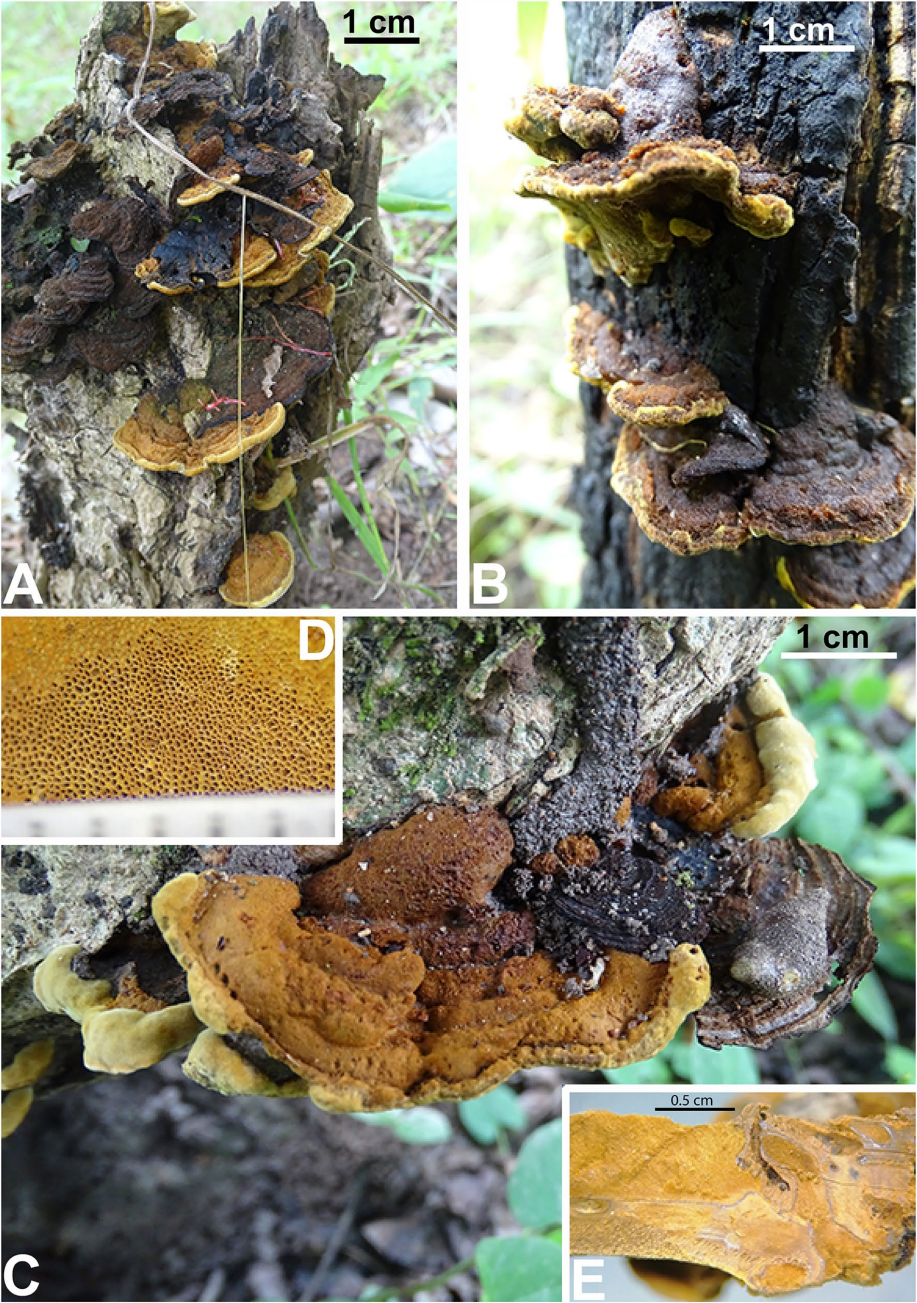
Macromorphology of *Phylloporia beninensis*. (**A**) Basidiomata on dead wood stump, (**B**) Basidiomata showing effused-reflexed attachment, (**C**) Basidiomata on dead part of living tree showing the margin of actively growing specimens, (**D**) poroid hymenophore, (**E**) Context showing the black line separating the tomentum and the lower context.

**Figure 5:**
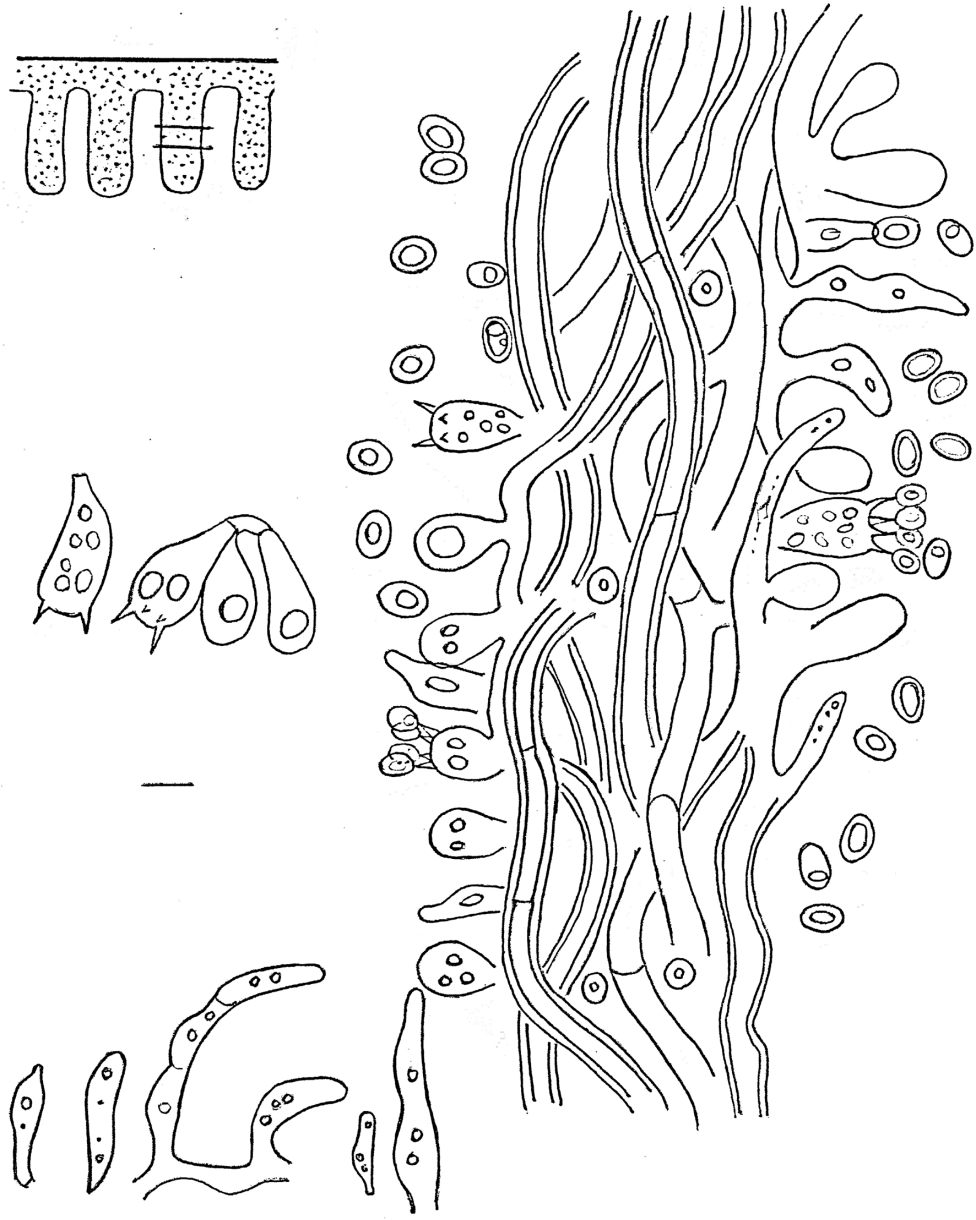
Line drawing of the hymenium of a pore of the type specimen of *Phylloporia beninensis* (OAB0511) showing the basidiospores, hyphae, basidia, basidioles, and cystidioles. Most elements with one or several guttulae. On the top left corner, we have the location where the microscopic preparation was taken. Scale bar = 5 μm.

**Figure 6:**
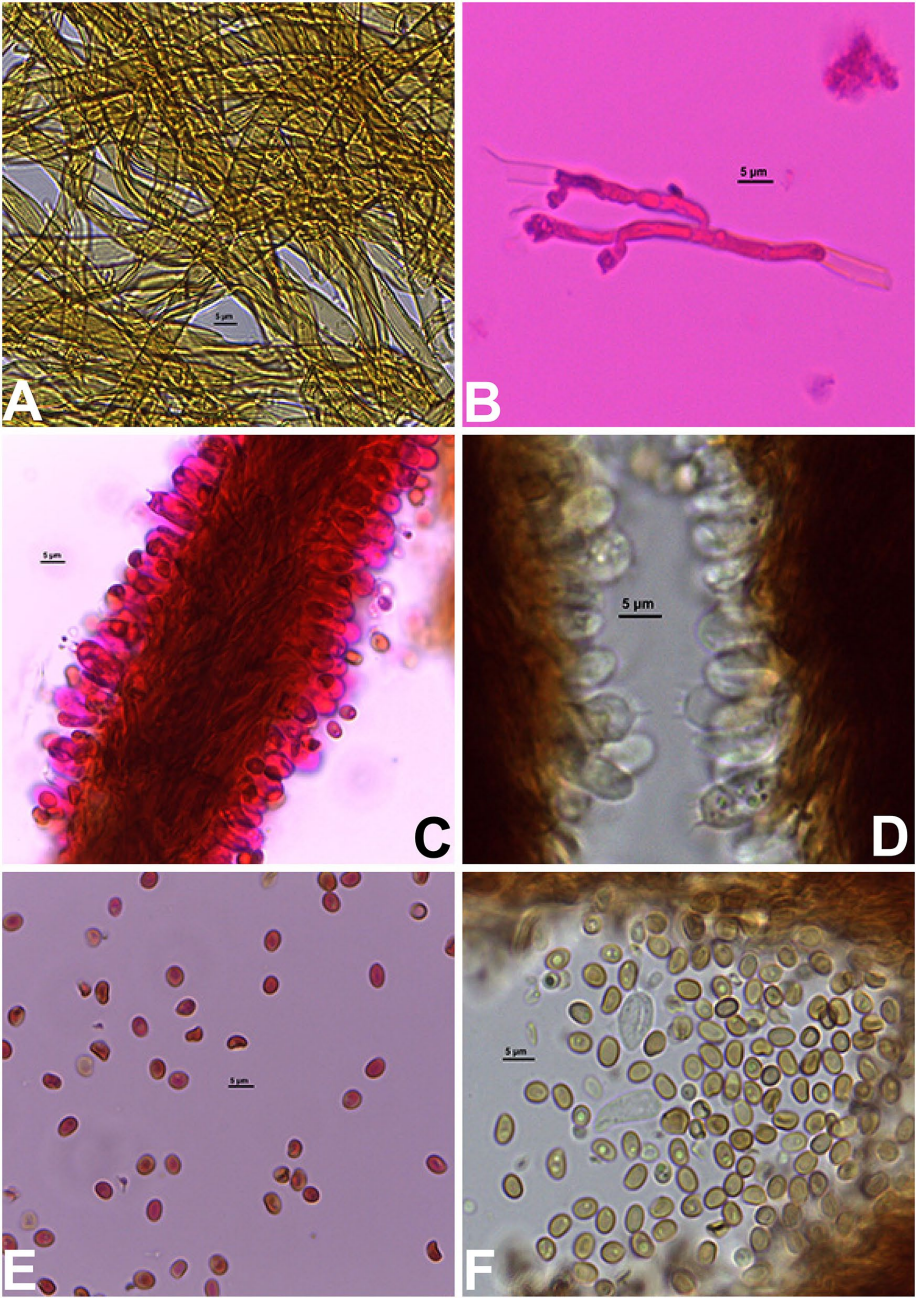
Microstructures of the type specimen of *Phylloporia beninensis*. (**A**) Hyphae from tomentum, (**B**) generative hyphae from trama in KOH mixed with 1% phloxine, (**C**) Section through the hymenium in KOH mixed with 1% phloxine showing basidia, basidioles, and basidospores, (**D**) section through the hymenium in KOH showing hyaline basidia and basidioles, some with several guttulae, (**E**) Basidiospores in KOH mixed with 1% phloxine, (**F**) Basidospores with one or two guttulae in KOH.

#### Diagnosis

*Phylloporia beninensis* differs from other known species of *Phylloporia* by the combination of the following characteristics: basidiomata imbricate; pileus projecting up to 3 cm, 5 cm wide, and 1 cm thick at base, surface concentrically sulcate and zonate; cystidioles present, variable in size and shape; basidia 9–12 × 4–5 μm; basidiospores ellipsoid to subglobose, 3–4.6 × 2.1–3.6 μm.

#### Holotype

BENIN. Borgou province, Woodlands of Okpara/Parakou, 9° 15′ 36.62″ N, 2° 43′ 28.40″ E, altitude 331 m.a.s.l., on dead stump of an unidentified angiosperm tree., leg. Boris A. Olou, sampling date: 11.09.2019, OAB0511 (dried specimen, holotype in UNIPAR and isotype in KAS). Holotype sequences: LSU, accession number: MW244096.

#### Etymology

*beninensis* (lat.): referring to the country of the type locality.

### Description

Basidiomata annual, pileate, sessile, imbricate with overlapping pilei, broadly attached or effused-reflexed (Fig. 4a–c), hard when dried, without odour or taste, projecting up to 3 cm, 5 cm wide, and 1 cm thick at the base. Pileus applanate to slightly convex, surface mustard and ferruginous brown in young or actively growing specimens and almost blackish in old specimens, velvety under stereomicroscope; surface concentrically sulcate and zonate; margin undulate, obtuse, yellowish when young or in actively growing specimens (Fig. 4c), concolorous with the pileus at maturity. Pore surface buff-yellow to honey, not shining or at least in the dried specimens, pore very small, 7–9 per mm, isodiametric to angular (Fig. 4d). Context two-layered, with a black line separating the upper context (tomentum) from the lower context, mustard brown, tomentum softer and lighter coloured than the lower context, tomentum up to 5 mm thick at the base and in the middle and thinner toward the margin, lower context up to 2 mm thick at the base and thinner at the margin (Fig. 4e). Tube layer concolorous with pore surface, up to 2 mm long.

Hyphal system dimitic (Fig. 5), skeletal hyphae of tomentum golden yellow in water, darker in KOH, thick-walled, unbranched, simple septate, interwoven, 3–6 μm in diam. (Fig. 6a). Skeletal hyphae in the lower context golden yellow in water, darker in KOH, thick-walled, unbranched, septate, 3–4 μm in diam., slightly interwoven. Trama with generative hyphae (Figs. 5, 6c); these hyaline, thin to thick-walled, occasionally branched, frequently simple septate, without clamp, 2–3 μm in diam. (Fig. 6b); skeletal hyphae abundant, dominating the trama, unbranched, septate, 3–4.5 μm in diam., thick-walled, wall thickness up to 1 μm, slightly interwoven to partially arranged.

Basidiospores normally abundant, smooth, with one or two guttulae, ellipsoid to subglobose, thin- to thick-walled, yellow–brown, hilar appendix nearly inevident (Figs. 5, 6e,f), inamyloid, acyanophilous, (3–)3.3–4.3(–4.6) × (2.1–)2.4–3.3(–3.6) µm, L = 3.8 μm, W = 2.8 μm, Q = 1.08–1.6 (n = 1088/1). Basidia tetrasterigmate; sterigmata up to 2.3 μm long, hyaline, clavate, 9–12 × 4–5 μm, with several guttulae; basidioles abundant, similar in shape to basidia, 9–11 × 4–6 μm, with several guttulae (Figs. 5, 6c,d). Cystidioles frequent, variable in size and shape.

#### Ecology and distribution

On deadwood or dead parts of living trees of woody angiosperms, including *Trichilia emetic*a Vahl. Currently known from the type locality and other localities of Benin.

#### Additional materials examined

BENIN. Collines province, woodlands of Kilibo/*Ouèssè*, leg. Boris A. Olou, on dead wood stump of *T. emetic*a, 17.08.2017, 8° 32′ 36.39″ N, 2° 41′ 12.80″ E, altitude 312 m.a.s.l., OAB0107 (UNIPAR); Borgou province, Ouémé Supérieur reserve forest, on dead part of an unidentified angiosperm living tree, 9° 45′ 29.09″ N, 2° 19′ 58.78″ E, altitude 334 m.a.s.l., 24.08.2017, OAB0142 (KAS).

## Discussion

Phylogenetic analyses inferred from the LSU and ITS-LSU datasets, coupled with macro- and microscopic examinations and ecological analyses, support the recognition of *P. beninensis* as a new species. *Phylloporia beninensis* is morphologically distinguished from other *Phylloporia* species by its annual, sessile, pileate, and imbricate basidiomata, two-layered context with the layers separated by a black line, dimitic hyphal system, and presence of cystidioles that vary in size and shape.

*Phylloporia beninensis* is macroscopically most similar to *P*. *rattanicola* F. Wu, G.J. Ren & Y.C. Dai; the two species share the pileate and imbricate basidiomata, velutinous pileus surface, two-layered context separated by a black line, presence of cystidioles, and dimitic hyphal system^8^. *Phylloporia rattanicola* differs from *P*. *beninensis* in its perennial basidiomata; smaller pores (9–11 per mm), and cyanophilic basidiospores^8^. *Phylloporia minutipora* L.W. Zhou is also similar in its annual, sessile basidioma with velutinate pileus surface, duplex context, and a dimitic hyphal system^41^. However, *P. minutipora* can be easily differentiated from *P*. *beninensis* by its much smaller pore size (12–15 per mm), larger basidiomata that project up to 10 cm from the substratum, absence of cystidioles, and smaller basidiospores 2.5–3 × 1.5–2.5 μm^41^. In addition to these morphological differences, *P. beninensis* clustered in a strongly supported and distinct lineage within *Phylloporia* clade in our molecular phylogenetic analyses (Figs. 2, 3). In these analyses *P. beninensis* has a strong phylogenetic relationship (PP = 1.00, BS = 95%) with an unidentified species of *Phylloporia* from Kenya (MUCLKE 16107, GenBank KY349147)^17^ and is phylogenetically distant from *P*. *rattanicola* and *P*. *minutipora*.

We cannot yet confirm whether or not *P. beninensis* is saprotrophic even though it was mainly found on dead wood (Fig. 4a,b), because it is well evidenced, that the habit of a fungus to produce fruit body on dead wood does not necessarily indicate a saprotrophic lifestyle^42^. However, although the lifestyle of *P. beninensis* is not yet well known, the fact that it was mainly found on dead wood we can reasonably say that the latter is saprotroph. As saprotroph, *P. beninensis* is therefore ecologically different from *P*. *minutipora* and *Phylloporia* sp., which are mainly collected from living trees^17, 41^. Like *P. beninensis*, *P. rattanicola* is also saprotrophic because it was collected from dead rattan^8^. However, knowing that *Phylloporia* species display a high level of host specificity^7, 10, 15, 43^, and that *P. rattanicola* is only collected on rattan while *P. beninensis* is collected on hardwood, we can safely say that *P. beninensis* and *P. rattanicola* do not belong to the same morpho- ecological group as stated above.

**Figure 7:**
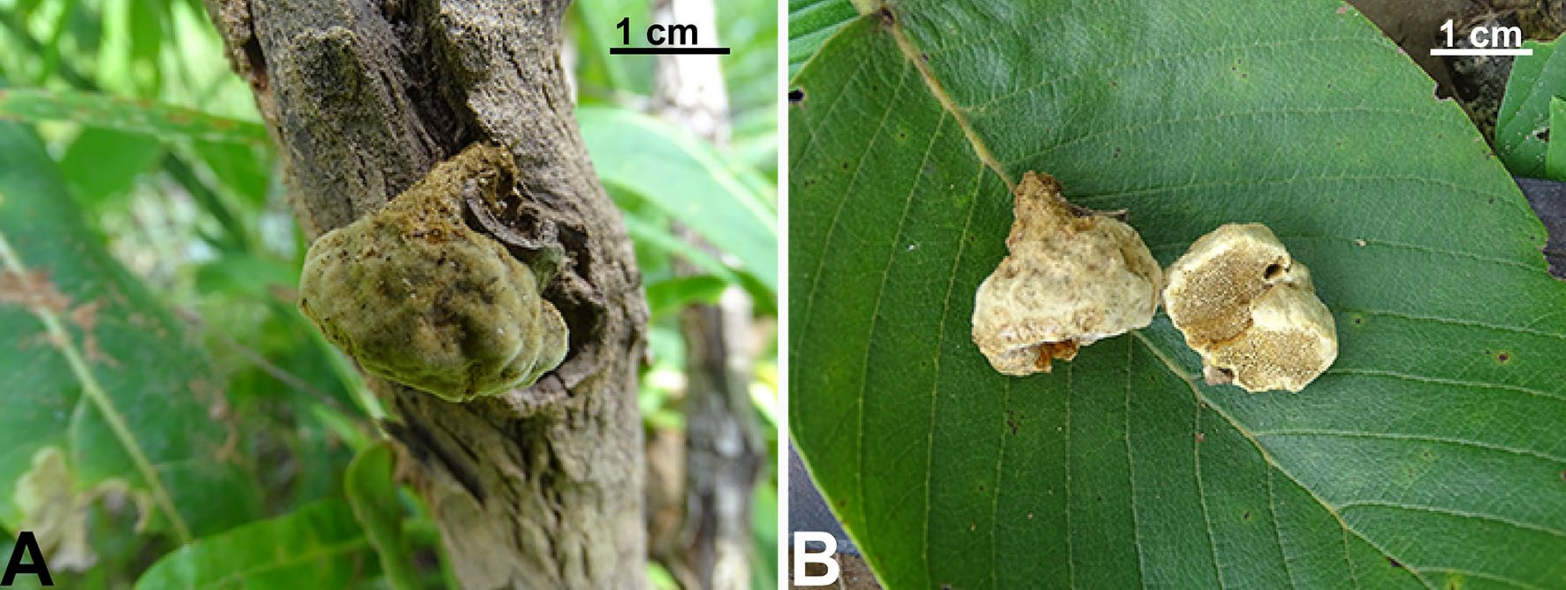
Field photos of *Phylloporia littoralis* (OAB0204). (**A**) Basidioma attached to a branch of…, (**B**) Pileus surface and hymenophore.

We also reported here *P. littoralis* Decock & Yombiyeni on the basis of molecular and morphological analyses, constituting the first record of the species from Benin (Figs. 2, 3, 7). The Benin *P. littoralis* specimen fits well morphologically and genetically to the Central African type specimen (see Fig. 2, in Yombiyeni and Decock 2017). To our knowledge, this is the first time *P. littoralis* has been reported outside its type locality Gabon, and suggests that the species may be more widely distributed in sub-Saharan Africa.

The recognition of *P. beninensis* brings the number of described *Phylloporia* species to 62 worldwide. Among these 62 species, nine were described from tropical Africa^9, 12, 17, 18, 23^. *Phylloporia* are more diverse in tropical Africa in comparison with Europe, where only *P. ribis* (Schumach.) Ryvarden has been reported^4^ to date. Considering that tropical Africa remains poorly explored for wood-decay fungi, it is likely that many more *Phylloporia* species remain to be found. We are also confident that new investigations of new still unexplored habitats and re-examination of herbarium specimens initially assigned to the genus *Phellinus* will reveal more new species of *Phylloporia* from tropical Africa. Aside the nine species described with type specimens, six other *Phylloporia* species have been reported from tropical Africa^2, 7^, which brings the number of regional *Phylloporia* species to 15. To facilitate future taxonomic studies in the genus, we provide a dichotomous key for identification of tropical African *Phylloporia* species.

Identification key to African *Phylloporia* species

1. Basidiomata resupinate on the underside of living leaves…***P. parasitica***
2. Basidiomata sessile to stipitate…2
3. Basidiomata stipitate…3
4. Basidiomata sessile…5
5. Context homogenous, black line lacking…***P. minutispora***
6. Context duplex, black line present…4
7. Pores 7–9 per mm…***P. spathulata***
8. Pores 10–11 per mm…***P. afrospathulata***
9. Perennial, pore surface glancing…***P. pectinata***
10. Annual, pore surface not glancing…6
11. Basidiomata gregarious…7
12. Basidiomata solitary to imbricate…9
13. Clustered in more than 100 individuals, pileus shiny…***P. flabelliformis***
14. Clustered in a small groups of less than 100 individuals, pileus dull…8
15. Hyphal system monomitic, pores 5–6 per mm…***P. gabonensis***
16. Hyphal system dimitic, pores 9–11 per mm…***P. fulva***
17. Cystidioles present…10
18. Cystidioles absent…11
19. Cystidioles fusoid, pores sinuous to subdaedaleoid, (1.5–) 2–3 per mm…***P. inonotoides***
20. Cystidioles variable in shape and size and up to 30 μm long, pores round to angular, 7–9 per mm…***P. beninensis***
21. On living trees and bushes…12
22. On dead and Q3living trees…14
23. Host specific, found on species of *Rinorea* (Violaceae)…***P. rinoreae***
24. Not host specific…13
25. Basidioma 0.5–3 cm in diam., 0.5–1 cm thick, basidia 8.5 × 5 µm…***P. littoralis***
26. Basidioma 1–5 cm in diam., up to 2 cm thick, basidia 8–10 × 3–4 µm…***P. fruticum***
27. Basidiospores 3–4.5 × 2.5–3.5 µm…***P. weberiana***
28. Basidiospores 2.5–3.5 × 2–2.5 μm…***P. pulla***

## Supplementary Information

Supplementary Information.

## Data Availability

Alignment and phylogenetic tree from the combined ITS-LSU dataset generated in this study are available in TreeBASE under this link: http://purl.org/phylo/treebase/phylows/study/TB2:S27303. Newly generated sequences are available in GenBank and the accession numbers are given in Table 1. Alignment, phylogenetic tree, and accession numbers of newly generated sequences will be public after the paper is published. Collected specimens are available at the mycological herbaria of the University of Parakou (UNIPAR) in Benin and University of Kassel (KAS) in Germany. Following the new requirement of MycoBank, the new species will be registered in MycoBank and the registration number will be given in the taxonomy section of this paper as soon as the paper is accepted.

## Abbreviations

ABGD: Automatic barcode gap detection
BS: Bootstrap values
BY: Bayesian
ITS: Internal transcribed spacer
KAS: Mycological Herbarium of the University of Kassel
L: Length
LSU: Large subunit
m a.s.l.: Meters above sea level
ML: Maximum likelihood
*nr*DNA: Nuclear ribosomal DNA
PP: Posterior probabilities
PTP: Poisson tree process
PTP_Bs: Poisson tree process Bayesian solution
PTP_Mls: Poisson tree process maximum likelihood solution
Q: Length to width ratio
UNIPAR: Mycological Herbarium of the University of Parakou, Benin
W: Width
